# Occurrence of *Toxoplasma gondii* and *Neospora caninum* Antibodies in Pet Cats and Dogs in Pathum Thani, Thailand

**DOI:** 10.3390/tropicalmed11040089

**Published:** 2026-03-25

**Authors:** Nhung Pho Nguyen Nguyen, Thuy Thi Nguyen, Chonchadayu Phanpha, Ketsarin Kamyingkird, Adrian B. Hehl, Tawin Inpankaew

**Affiliations:** 1 Department of Parasitology, Faculty of Veterinary Medicine, Kasetsart University, Bangkok 10900, Thailand; nhungphonguyen.n@ku.th (N.P.N.N.); darlene_vetmed@hotmail.com (C.P.); ketsarin.ka@ku.th (K.K.); 2 Department of Veterinary Medicine, Faculty of Animal Science and Veterinary Medicine, College of Agriculture and Forestry, Hue University, Hue City 530000, Vietnam; nguyenthithuy@huaf.edu.vn; 3 Laboratory of Molecular Parasitology, Institute of Parasitology, Vetsuisse and Medical Faculty, University of Zurich, CH-8057 Zurich, Switzerland; adrian.hehl@uzh.ch; 4 One Health Institute, Vetsuisse, Science, and Medical Faculties, University of Zurich, CH-8057 Zurich, Switzerland

**Keywords:** *Toxoplasma gondii*, *Neospora caninum*, Indirect fluorescent antibody test, Pathum Thani

## Abstract

*Toxoplasma gondii* and *Neospora caninum* are closely related apicomplexan parasites of veterinary and public health importance. *T. gondii* is a zoonotic pathogen for which cats are the definitive host, whereas *N. caninum* is a major cause of reproductive losses in cattle, with dogs acting as the definitive host. Data on exposure in pet animals in Thailand remain limited. This study investigated seroprevalence and associated risk factors of *T. gondii* and *N. caninum* in pet cats and dogs in Pathum Thani Province, an urban area adjacent to Bangkok. Between June 2020 and July 2021, serum samples were collected from 169 owned animals, including 86 cats and 83 dogs, participating in a mobile sterilization program. Antibodies were detected using the indirect fluorescent antibody test (IFAT), and animal characteristics, behaviors, and environmental factors were obtained via owner questionnaires. Serological evidence of exposure to both parasites was detected. Antibodies against *T. gondii* were detected in 4.73% (8/169) of animals, including 4.65% (4/86) of cats and 4.82% (4/83) of dogs. For *N. caninum*, the overall seroprevalence was 10.06% (17/169), with a higher prevalence in dogs (15.66%, 13/83) than in cats (4.65%, 4/86). No significant risk factors were identified for *T. gondii* or *N. caninum* infection in either cats or dogs (*p* > 0.05).

## 1. Introduction

Pet animals, particularly dogs and cats, play an important role in human society by providing companionship and emotional support. During periods of social disruption, such as the COVID-19 pandemic, close human–animal relationships have been shown to contribute positively to mental health and well-being [1]. At the same time, dogs and cats can serve as hosts for several parasitic pathogens, including *Toxoplasma gondii*, a zoonotic parasite of public health concern, and *Neospora caninum*, which is primarily of veterinary importance. Through the shedding of environmentally resistant oocysts, these parasites can contaminate soil, water, and food sources, thereby facilitating transmission to other animals and humans [2,3].

In Thailand, large populations of free-roaming and owned animals coexist in urban and peri-urban environments. Stray and semi-owned dogs and cats are commonly observed in public spaces such as temples, schools, streets, and residential areas, often in close proximity to humans and household pets [4]. This close contact creates opportunities for pathogen circulation between animals and people highlighting the importance of monitoring zoonotic and veterinary parasites in companion animals.

*T. gondii* and *N. caninum* are obligate intracellular protozoa belonging to the phylum Apicomplexa and share close genetic and biological similarities [5]. *N. caninum* is recognized as a major cause of abortion and reproductive failure in cattle, leading to substantial economic losses in livestock production worldwide [6,7,8,9]. Dogs act as the definitive host of *N. caninum*, shedding oocysts into the environment. In contrast, *T. gondii* is a globally distributed zoonotic parasite capable of infecting all warm-blooded animals, including humans, with cats serving as the definitive host [3]. Infections acquired during pregnancy, particularly in humans and livestock, can result in severe outcomes such as miscarriage, stillbirth, or neonatal disease [10].

Both parasites have heteroxenous life cycles involving sexual reproduction in definitive hosts and asexual multiplication in intermediate hosts [2]. Dogs and cats may become infected through ingestion of sporulated oocysts from contaminated environments, consumption of raw or undercooked meat containing tissue cysts, or through vertical transmission from an infected dam to her offspring [3,10]. As a result, exposure risk is influenced by animal behavior, management practices, and environmental conditions.

Several studies have reported seroprevalence of *T. gondii* and *N. caninum* in dogs and cats in Thailand, although available data remain limited. In Thailand, seroprevalence of *T. gondii* in dogs has been reported to range from approximately 8% to 11% [11,12,13], while in cats it varies more widely, from 5% to over 30% depending on population characteristics such as owned or stray status [11,14,15]. For *N. caninum*, seroprevalence in dogs has been reported at relatively low levels, and data on exposure in cats are scarce [16,17]. Importantly, information on the concurrent seroprevalence of both parasites in pet dogs and cats, as well as associated risk factors, is lacking for Pathum Thani Province, an urban area adjacent to Bangkok.

Therefore, the objective of the present study was to determine the seroprevalence of *T. gondii* and *N. caninum* in pet cats and dogs in Khukhot City Municipality, Pathum Thani Province, Thailand, using the indirect fluorescent antibody test (IFAT). In addition, selected animal- and environment-related variables were evaluated to identify potential risk factors associated with exposure to these parasites. The findings aim to contribute to a better understanding of parasite circulation in urban pet populations and to inform preventive measures relevant to animal and public health.

## 2. Materials and Methods

### 2.1. Ethical Statement

All procedures involving animals were conducted in accordance with ethical standards and with the informed consent of animal owners. Sample collection was performed by licensed veterinarians as part of a mobile sterilization program in Khukhot City Municipality, Pathum Thani Province, Thailand.

### 2.2. Animals and Blood Collection

Sample collection was carried out between June 2020 and July 2021 as part of a mobile sterilization program in Khukhot City Municipality, Pathum Thani Province, Thailand. Blood samples were obtained from owned dogs and cats whose owners provided verbal consent. A total of 169 serum samples were collected, comprising 83 dogs and 86 cats. It should be noted that the study population represents a convenience sample of animals presented to the mobile sterilization program and may not be fully representative of the broader pet population in Pathum Thani Province.

Blood samples were collected from the jugular vein in cats and from the cephalic vein in dogs and transferred into sterile tubes without anticoagulant. Samples were allowed to clot at room temperature for approximately 30–60 min prior to centrifugation. Samples were then centrifuged at 1100× *g* for 10 min to separate serum, which was then stored at −20 °C until serological analysis.

Animal owners were interviewed using a structured questionnaire to obtain metadata information on animal-related and environmental variables, including sex, age, breed, behavior, and housing or environmental conditions. Age groups were categorized as <6 months, 6–12 months, 1–7 years, and >7 years. These data were used for subsequent risk factor analysis in both dogs and cats.

### 2.3. Serological Assay

Detection of antibodies against *T. gondii* and *N. caninum* in dog and cat sera was performed using the indirect fluorescent antibody test (IFAT), following previously described protocols with minor modifications [18,19].

*T. gondii* (RH strain) and *N. caninum* (NC-1 strain) tachyzoites were propagated in African green monkey kidney (Vero) cell cultures maintained in minimum essential medium (MEM; Sigma-Aldrich, St. Louis, MO, USA) supplemented with 10% heat-inactivated fetal bovine serum, L-glutamine, and penicillin–streptomycin at 37 °C in a humidified atmosphere containing 5% CO_2_. Tachyzoites were harvested by repeated passage through syringe needles and filtration through a 5 µm filter, followed by three washes in phosphate-buffered saline (PBS).

Parasites were adjusted to final concentrations of 1 × 10^6^ tachyzoites/mL for *T. gondii* and 1 × 10^5^ tachyzoites/mL for *N. caninum*. Teflon-coated 12-well slides were prepared by dispensing 10 µL of tachyzoite suspension into each well. Slides were air-dried at room temperature, fixed in acetone for 30 min at room temperature, and stored at −20 °C until use.

Test sera were serially diluted twofold starting at a dilution of 1:50 and incubated on antigen-coated slides at 37 °C for 30 min. Slides were then washed with PBS containing 4% bovine serum albumin. Bound antibodies were detected using fluorescein isothiocyanate (FITC)-conjugated caprine anti-feline IgG or anti-canine IgG (VMRD, Washington, DC, USA), as appropriate. Slides were examined under a fluorescence microscope after mounting with cover slips.

All slides were independently evaluated by two experienced investigators who were blinded to the sample identity. A sample was considered positive only when clear, complete peripheral fluorescence outlining the entire tachyzoite was observed. Samples showing only partial, diffuse, or apical fluorescence (polar staining) were considered negative to minimize false-positive interpretation. In cases of discrepant results, slides were re-evaluated jointly and a consensus was reached.

Serum samples were tested for antibodies against *T. gondii* and *N. caninum* using the indirect fluorescent antibody test (IFAT). A serum dilution of ≥1:100 was considered positive for both parasites. This threshold has been applied in several previous IFAT-based seroepidemiological studies in dogs and cats and is commonly used to increase diagnostic specificity and reduce potential non-specific fluorescence at lower dilutions [20,21].

### 2.4. Statistical Analysis

Seroprevalence of *T. gondii* and *N. caninum* was calculated as the proportion of seropositive animals among the total number of animals tested. Associations between seropositivity and categorical variables, including age, sex, breed, behavior, and environmental conditions, were evaluated using the Chi-square test. When expected cell counts were less than 5, Fisher’s exact test was applied instead. Statistical significance was defined as *p* < 0.05.

## 3. Results

### 3.1. Study Population

A total of 169 serum samples were collected from owned pet animals in Khukhot City Municipality, Pathum Thani Province, Thailand, comprising 86 cats and 83 dogs. Animals included both sexes and a range of age groups, as defined in the questionnaire-based survey.

### 3.2. Seroprevalence of T. gondii

Antibodies against *T. gondii* were detected in 8/169 (4.73%) animals examined using an IFAT cut-off of ≥1:100. Seroprevalence was similar between species, with 4/86 (4.65%) cats and 4/83 (4.82%) dogs testing seropositive (Table 1). Most seropositive animals exhibited moderate antibody titers, predominantly in the range of 1:100 to 1:200, with higher titers observed in a small number of cases.

In dogs, seroprevalence was similar between males and females. The highest proportion of seropositive dogs was observed in animals aged 1–7 years. In cats, seropositivity was also most frequently detected in the 1–7 years age group. Although the seroprevalence in male cats was approximately three times higher than that in females, this difference was not statistically significant. Overall, no statistically significant associations were identified between *T. gondii* seropositivity and sex or age in either cats or dogs (*p* > 0.05) (Table 2 and Table 3).

### 3.3. Seroprevalence of N. caninum

Antibodies against *N. caninum* were detected more frequently than those against *T. gondii*. Overall, 10.06% (17/169) of the sampled animals were seropositive for *N. caninum*. A markedly higher seroprevalence was observed in dogs (15.66%) compared with cats (4.65%) (Table 4). Antibody titers against *N. caninum* ranged from 1:100 to 1:800. Most seropositive animals showed low antibody titers, with 11 out of 17 positive samples detected at a titer of 1:100. Higher titers (1:200–1:800) were observed in a subset of animals, and no titers above 1:800 were detected.

Seroprevalence of *N. caninum* was similar between males and females in both dogs and cats. The highest proportion of seropositive animals was observed in the 6–12 months age group in both species. Nevertheless, no statistically significant associations were identified between *N. caninum* seropositivity and sex or age in either dogs or cats (*p* > 0.05) (Table 5 and Table 6).

### 3.4. Summary of Risk Factor Analysis

Statistical analysis did not reveal statistically significant associations between seropositivity for either parasite and the evaluated host-related variables, including sex and age, in cats or dogs. While variation in seroprevalence was observed across demographic categories, these differences did not reach statistical significance and should therefore be interpreted cautiously.

## 4. Discussion

This study provides serological evidence of exposure to *T. gondii* and *N. caninum* among pet cats and dogs in Khukhot City Municipality, Pathum Thani Province, Thailand. The seroprevalence of *T. gondii* was relatively low and comparable between cats and dogs. Antibodies against *N. caninum* were also detected in both species, although the distribution of antibody titers suggested that most seropositive animals had relatively low antibody levels.

The relatively low seroprevalence of *T. gondii* observed in this study is consistent with previous reports from Thailand and other urban settings, where owned animals typically have limited exposure to contaminated environments or raw meat [11,12,13,14,22,23,24,25]. Seroprevalence was comparable between cats (4.65%) and dogs (4.82%), suggesting that both species may be exposed to similar environmental sources of infection within the study area. Although cats are the definitive hosts of *T. gondii*, this comparable prevalence likely reflects shared environmental exposure rather than species-specific transmission dynamics.

In contrast, antibodies against *N. caninum* were detected more frequently than those against *T. gondii*, with an overall seroprevalence of 10.06%. A markedly higher prevalence was observed in dogs compared with cats, which is consistent with the recognized role of dogs as the definitive host of *N. caninum*. Although seroprevalence in cats was lower, the detection of antibodies indicates that cats can be exposed to *N. caninum* under natural conditions.

In Thailand, previous studies have reported relatively low seroprevalence of *N. caninum* in dogs, generally at lower than that observed in the present study [16,17]. In comparison, studies from other countries have reported more variable seroprevalence estimates [25,26,27,28,29,30], likely influenced by differences in geography, host populations, and diagnostic approaches [31]. Reports of *N. caninum* exposure in cats are comparatively rare, and seropositivity in this species is often interpreted cautiously, as cats are not considered definitive hosts. Nevertheless, experimental and field studies have demonstrated that cats are susceptible to *N. caninum* infection and can develop detectable antibody responses [20,25,32]. Therefore, seropositivity in cats observed in this study likely reflects environmental exposure rather than a significant role in parasite transmission.

Most seropositive animals exhibited low to moderate antibody titers, with higher titers observed in a small number of cases, suggesting subclinical or past exposure rather than active disease. However, interpretation of antibody titers in IFAT is limited, as serological results reflect exposure rather than infection status. Therefore, it is not possible to determine whether higher titers indicate recent or active infection in the absence of additional diagnostic evidence.

No statistically significant associations were identified between seropositivity and sex or age (*p* > 0.05), which may reflect the relatively small number of seropositive animals and the resulting limited statistical power. Consequently, the ability to detect meaningful associations in the risk factor analysis was constrained. The detection of antibodies in younger animals may also raises the possibility of vertical transmission, particularly for *N. caninum*, although this study was not designed to assess transmission routes directly.

From a One Health perspective, improved public awareness regarding appropriate feeding practices for cats may reduce hunting behavior and exposure to tissue-borne parasites. This is particularly relevant for *T. gondii*, a zoonotic parasite transmitted to humans via ingestion of oocysts shed by cats or through contaminated food and water. In contrast, *N. caninum* is not considered a major zoonotic pathogen but remains important in animal health, particularly in cattle. Therefore, improved awareness and surveillance may help reduce environmental contamination and support both animal and public health.

Several limitations should be considered. First, the study population represents a convenience sample from a mobile sterilization program and may not be representative of the broader pet population in Pathum Thani Province. Second, serological interpretation using IFAT is inherently subjective and may vary depending on the experience of the observer, despite efforts to standardize reading criteria. Finally, further studies using larger sample sizes and complementary diagnostic methods would be useful to better clarify the epidemiological significance of these findings.

## 5. Conclusions

This study provides serological evidence of exposure to *T. gondii* and *N. caninum* among pet cats and dogs in Khukhot City Municipality, Pathum Thani Province, Thailand. A relatively low seroprevalence of *T. gondii* was detected, with similar rates observed in cats and dogs. In contrast, *N. caninum* antibodies were detected more frequently, particularly in dogs, consistent with their role as definitive hosts of the parasite. Most seropositive animals exhibited low to moderate antibody titers, suggesting previous exposure rather than active infection. These findings contribute to the limited epidemiological data on these parasites in companion animals in Thailand and highlight the need for further studies with larger sample sizes and complementary diagnostic approaches to better understand transmission dynamics and potential public and animal health implications.

## Figures and Tables

**Table 1:** Seroprevalence of *T. gondii* antibodies in pet cats and dogs from Khukhot City Municipality, Pathum Thani Province, Thailand, using IFAT cut-off titers (≥1:100) including antibody titer distribution.

*Toxoplasma gondii*	Number of Samples	≥1:100 (*n*, %)	Titer					
			100	200	400	800	1600	3200
Cat	86	4 (4.65)	2	1	-	-	-	1
Dog	83	4 (4.82)	1	2	-	1	-	-
Total	169	8 (4.73)	3	3	-	1	-	1

**Table 2:** Seroprevalence of *T. gondii* antibodies in dogs from Khukhot City Municipality, Pathum Thani Province, Thailand, stratified by sex and age.

Parameter	IFAT+/Total (%)	*p*-Value
Sex		1.00
Male	2/42 (4.76%)	
Female	2/41 (4.88%)	
Age		0.55
<6 months	0/4 (0%)	
6–12 months	1/11 (9.09%)	
1–7 years	3/44 (6.82%)	
>7 years	0/24 (0%)	
Total	4/83 (4.82%)	

**Table 3:** Seroprevalence of *T. gondii* antibodies in cats from Khukhot City Municipality, Pathum Thani Province, Thailand, stratified by sex and age.

Parameter	IFAT+/Total (%)	*p*-Value
Sex		0.34
Male	3/41 (7.32%)	
Female	1/45 (2.22%)	
Age		1.00
<6 months	0/10 (0%)	
6–12 months	1/23 (4.35%)	
1–7 years	4/49 (6.12%)	
>7 years	0/4 (0%)	
Total	4/86 (4.65%)	

**Table 4:** Seroprevalence of *N. caninum* antibodies in pet cats and dogs from Khukhot City Municipality, Pathum Thani Province, Thailand, using IFAT cut-off titers (≥1:100), including antibody titer distribution.

*Neospora caninum*	Number of Samples	≥1:100 (*n*, %)	Titer					
			100	200	400	800	1600	3200
Cat	86	4 (4.65)	2	1	-	1	-	-
Dog	83	13 (15.66)	9	1	1	2	-	-
Total	169	17 (10.06)	11	2	1	3	-	-

**Table 5:** Seroprevalence of *N. caninum* antibodies in dogs from Khukhot City Municipality, Pathum Thani Province, Thailand, stratified by sex and age.

Parameter	IFAT+/Total (%)	*p*-Value	χ ^2^	df
Sex		0.79	0.06	1
Male	7/42 (16.67%)			
Female	6/41 (14.63%)			
Age		0.57	2.02	3
<6 months	0/4 (0.00%)			
6–12 months	3/11 (27.27%)			
1–7 years	6/44 (13.64%)			
>7 years	4/24 (16.67%)			
Total	13/83 (15.66%)			

**Table 6:** Seroprevalence of *N. caninum* antibodies in cats from Khukhot City Municipality, Pathum Thani Province, Thailand, stratified by sex and age.

Parameter	IFAT+/Total (%)	*p*-Value
Sex		1.00
Male	2/41 (4.88%)	
Female	2/45 (4.44%)	
Age		0.24
<6 months	0/10 (0.00%)	
6–12 months	3/23 (13.04%)	
1–7 years	1/49 (2.04%)	
>7 years	0/4 (0.00%)	
Total	4/86 (4.65%)	

## Data Availability

The original contributions presented in this study are included in the article. Further inquiries can be directed to the corresponding author.

## Abbreviations

The following abbreviations are used in this manuscript:

IFAT	Indirect fluorescent antibody test
MEM	Minimum essential medium
PBS	Phosphate-Buffered Saline
